# Clinical and immunological features of prolonged and chronic endogenous manic and manic-delusional states in the structure of endogenous diseases

**DOI:** 10.1192/j.eurpsy.2024.1429

**Published:** 2024-08-27

**Authors:** S. A. Zozulya, E. B. Rumyantseva, S. V. Sizov, I. V. Oleichik, T. P. Klyushnik

**Affiliations:** Mental Health Research Centre, Moscow, Russian Federation

## Abstract

**Introduction:**

The relevance of studying the clinical and immunological characteristics of prolonged and chronic endogenous manic and manic-delusional states is conditioned by their high prevalence, insufficient understanding of pathogenetic mechanisms, and the need to develop adequate therapeutic approaches.

**Objectives:**

To study the clinical and biological correlations between inflammatory markers of blood plasma, the severity of manic symptoms and psychopathological characteristics of patients with prolonged and chronic endogenous manic and manic-delusional states.

**Methods:**

70 female patients aged 18 to 55 years (mean age 33.6±5.9 years) with prolonged and chronic endogenous manic and manic-delusional states within different nosologies (F31.1-2, F25.01, F25) were examined. Psychometric assessment was performed using the PANSS, YMRS, and GAF scales. The control group consisted of 55 mentally and somatically healthy women of the corresponding age.

Leukocyte elastase (LE) activity, α1-proteinase inhibitor (α1-PI) activity, and the autoantibody levels to astrocytic protein S-100B and myelin basic protein (MBP) were determined in blood plasma.

**Results:**

The increase in the level of immune system activation of different degrees (according to the complex of inflammatory and autoimmune markers) associated with the severity of the patient’s condition within the examined nosologies was revealed.

The highest level of immune activation, characterized by an increase in the activity of both LE and α1-PI (p<0.01), and the level of autoantibodies to S-100B and MBP (p<0.05), was characteristic of patients with chronic endogenous manic and manic-delusional states in the framework of schizophrenia. Manic symptoms within different nosologies had clinical features, however, no differences in the severity of these symptoms on the YMRS scale were revealed (p>0.05).

Positive correlations were found between LE activity and the PANSS subscale of general psychopathological symptoms (R=0.3, p=0.006) and the PANSS total score (R=0.3, p=0.03). The level of antibodies to S-100B correlated with the PANSS negative subscale score (R=0.3, p=0.04). A negative correlation was found between LE activity and the level of social functioning of patients according to the GAF scale (R=-0.3, p=0.02).

**Conclusions:**

The immune profile of patients with prolonged and chronic manic and manic delusional states within endogenous psychiatric disorders is determined mainly by nosologic affiliation, which is also related to the clinical features of manic states.

**Disclosure of Interest:**

None Declared

